# Important role of the SDF-1/CXCR4 axis in the homing of systemically transplanted human amnion-derived mesenchymal stem cells (hAD-MSCs) to ovaries in rats with chemotherapy-induced premature ovarian insufficiency (POI)

**DOI:** 10.1186/s13287-022-02759-6

**Published:** 2022-02-23

**Authors:** Li Ling, Jiying Hou, Dandan Liu, Dongyuan Tang, Yanqin Zhang, Qianru Zeng, Heng Pan, Ling Fan

**Affiliations:** 1 Department of Obstetrics and Gynecology, The Second Affiliated Hospital of Chongqing Medical University, No. 74, Linjiang Road, Chongqing, 400010 China; 2 Department of Otolaryngology, The Ninth People’s Hospital of Chongqing, Chongqing, 400700 China; 3 Department of Obstetrics and Gynecology, Wushan County People’s Hospital of Chongqing, Chongqing, 404700 China

**Keywords:** Premature ovarian insufficiency (POI), Human amnion-derived mesenchymal stem cells (hAD-MSCs), Transplantation, Stem cell homing, Stromal cell-derived factor-1 (SDF-1), CXC chemokine receptor 4 (CXCR4)

## Abstract

**Background:**

Chemotherapy can induce premature ovarian insufficiency (POI). POI causes multiple sequelae and is currently incurable. As shown in our previous studies, systemically transplanted human amnion-derived mesenchymal stem cells (hAD-MSCs) home to ovaries with chemotherapy-induced POI and subsequently reduce ovarian injury and improve ovarian function in rats with POI. However, the cellular mechanisms that direct the migration and homing of hAD-MSCs to ovaries with chemotherapy-induced POI are incompletely understood. This study investigated the role of the SDF-1/CXCR4 axis in the migration and homing of systemically transplanted hAD-MSCs to ovaries with chemotherapy-induced POI and its relevant downstream signalling pathways.

**Methods:**

CXCR4 expression in hAD-MSCs was assessed using Western blotting and immunofluorescence staining. hAD-MSC migration was tested using Transwell migration assays. SDF-1 levels were detected using ELISA. Seventy-two female SD rats were randomly divided into the control, POI, hAD-MSCs and hAD-MSCs + AMD3100 groups. Cyclophosphamide was used to establish rat POI models. For inhibitor treatment, hAD-MSCs were pretreated with AMD3100 before transplantation. PKH26-labeled hAD-MSCs were injected into the tail vein of POI rats 24 h after chemotherapy. After hAD-MSC transplantation, the homing of hAD-MSCs to ovaries and ovarian function and pathological changes were examined. We further investigated the molecular mechanisms by detecting the PI3K/Akt and ERK1/2 signalling pathways.

**Results:**

hAD-MSCs expressed CXCR4. SDF-1 induced hAD-MSC migration in vitro. SDF-1 levels in ovaries and serum were significantly increased in rats with chemotherapy-induced POI, and ovaries with POI induced the homing of hAD-MSCs expressing CXCR4. Blocking the SDF-1/CXCR4 axis with AMD3100 significantly reduced the number of hAD-MSCs homing to ovaries with POI and further reduced their efficacy in POI treatment. The binding of SDF-1 to CXCR4 activated the PI3K/Akt signalling pathway, and LY294002 significantly inhibited hAD-MSC migration induced by SDF-1 in vitro. Moreover, inhibition of the PI3K/Akt signalling pathway significantly reduced the number of systemically transplanted hAD-MSCs homing to chemotherapy-induced ovaries in rats with POI.

**Conclusions:**

SDF-1/CXCR4 axis partially mediates the migration and homing of systemically transplanted hAD-MSCs to the ovaries of rats with chemotherapy-induced POI, and the PI3K/Akt signalling pathway might be involved in the migration and homing of hAD-MSCs mediated by the SDF-1/CXCR4 axis.

## Background

Premature ovarian insufficiency (POI) is a clinical syndrome defined by a loss of ovarian activity before the age of 40 years [1]. Untreated POI may cause multiple sequelae, such as infertility, osteoporosis, increased risks of fracture and cardiovascular disease, depression, anxiety, and gynaecological issues [1]. POI can be caused by various factors, mainly including genetic, autoimmune, and iatrogenic factors. POI is caused by iatrogenic factors, including chemotherapy, in approximately 25% of patients [4]. Approximately 30% of women before the age of 35 years and 50% of women between the ages of 35 and 40 years who received chemotherapy are diagnosed with POI [2]. POI is irreversible and currently incurable. Regenerative medicine studies have suggested that mesenchymal stem cell (MSC) transplantation may represent an effective treatment for POI [3–5]. MSCs have been shown to have significant potential for clinical use. This clinical utility is due to their self-renewal capacity, convenient isolation, low immunogenicity permitting allogenic or xenogeneic transplantation, and potential to differentiate into tissue-specific cell types and promote vascularization [6–8]. Human amnion-derived mesenchymal stem cells (hAD-MSCs) have been proven to not only have the features of MSCs but also have unique merits for clinical utility [9–11]. As shown in our previous studies, some systemically transplanted hAD-MSCs migrate and home to the ovaries of rats with chemotherapy-induced POI, further reducing ovarian injury and improving ovarian function [8]. However, the mechanisms that direct the migration and homing of hAD-MSCs to ovaries with chemotherapy-induced POI are poorly understood.

MSC homing is defined as the arrest of MSCs within the vasculature of a tissue followed by transmigration across the endothelium [12]. Cell migration, an important component of cell homing, is defined as the movement of cells from the source to the region where a response or action is required [13, 14]. Exogenous MSC homing is a process in which transplanted MSCs are recruited from the peripheral bloodstream and migrate to injured or pathological tissue [15]. Efficient homing of stem cells is a prerequisite for the successful engraftment of transplanted MSCs. Any improvement of existing cell-based therapeutic approaches will depend on a better understanding of the interaction of stem cells with the environment that leads to homing and tissue integration [16]. Although the molecular mechanisms that direct the migration and homing of MSCs are only partially understood [16], numerous studies have shown that two important detailed mechanisms underlying the efficiency of MSC homing might be involved [17]. One is that specific ligands or receptors that are upregulated in injured tissues facilitate the trafficking, adhesion, and infiltration of MSCs. The other is that chemokine receptors, selectins and integrins expressed on MSCs are involved in the migration of MSCs across the endothelium and homing to injured tissues.

Chemokines are 8- to 12-kDa peptides that are involved in cell migration and homing [18]. Stromal cell-derived factor-1 (SDF-1) is a member of the CXC chemokine subfamily. SDF-1 is considered the most potent chemoattractive signal and has been identified as a stem cell homing factor [19]. SDF-1 is expressed by a wide variety of cells and is substantially upregulated in many tissues induced by “stress,” such as injury, inflammation, irradiation or chemotherapy [18–20]. A local increase in SDF-1 levels in the injured tissue is capable of recruiting stem cells to the site of injury, where they support tissue repair and regeneration [19]. Many studies have proposed that SDF-1 is one of the most important chemokines and homing factors for stem cells that promotes the migration and homing of stem cells to injured tissues [19, 21, 22]. According to previous studies [23], the chemokine receptor repertoire of human bone marrow-derived mesenchymal stem cells (BM-MSCs) determines their migratory activity, and harnessing the migratory potential of MSCs by modulating their chemokine-chemokine receptor interaction may be a powerful method to enhance the homing capacity of stem cells after transplantation. CXC chemokine receptor 4 (CXCR4), a G protein-coupled receptor, is the primary chemokine receptor for SDF-1. CXCR4 is widely expressed on various cells, including haematopoietic cells, endothelial and epithelial cells, neuronal cells, progenitor cells and stem cells [18]. These cells express CXCR4 and migrate along SDF-1 gradients [18]. A number of studies have revealed that locally elevated levels of SDF-1, acting through its specific receptor CXCR4 on MSCs, are critical for MSCs homing to injured tissue, including bone marrow, infarcted myocardium, traumatic brain and injured liver [19, 24, 25]. The SDF-1/CXCR4 axis is associated with the migration and homing of MSCs in vivo [26].

Systemically transplanted MSCs migrate and home to injured tissues and promote tissue regeneration. SDF-1 plays a role in the migration and homing of CXCR4-positive MSCs to injured tissue and regulates repair activity [27, 28]. The migration of MSCs is mediated by upregulated SDF-1 in injured tissue and its specific receptor CXCR4 expressed on MSCs [29, 30]. The mobilization and homing of MSCs are regulated by the interaction between SDF-1 and CXCR4 [19, 24, 25]. Increasing evidence supports the central role of the SDF-1/CXCR4 axis in regulating the migration and homing of MSCs to injured tissue. Thus, in this study, we explored whether the SDF-1/CXCR4 axis is involved in the migration and homing of systemically transplanted hAD-MSCs to the ovaries of rats with chemotherapy-induced POI.

Binding of SDF-1 to CXCR4 activates multiple signalling pathways to regulate cell migration, proliferation, differentiation, survival and apoptosis [26, 31]. However, the signalling networks connecting the SDF-1/CXCR4 axis with cell migration and homing are still not well understood. Signalling cascades induced by SDF-1/CXCR4 activate related pathways in stem cells, including p44/p42 extracellular signal-regulated kinases (ERK1/2) and phosphatidylinositol-3-kinase (PI3K)/Akt, which affect the chemotaxis and migration of stem cells and result in stem cell mobilization from the bone marrow into the peripheral blood, as well as their homing to the injured tissue [19, 32]. CXCR4-mediated chemotaxis is mediated by the activation of PI3K [18]. PI3K activation results in the phosphorylation and activation of several downstream components, such as Akt [18]. PI3K/Akt activation participates in the migration of MSCs, and the CXCR4 antagonist AMD3100 reverses the increase in the phospho-Akt level and inhibits MSC migration induced by the SDF-1/CXCR4 axis [33, 34]. Additionally, chemotaxis mediated by the SDF-1/CXCR4 axis may be driven by the activation of MAPK through ERK1/2 [19, 35]. The SDF-1/CXCR4 axis mediates MSC migration, and the activation of the ERK 1/2 downstream signalling pathway induced by the binding of SDF-1 to CXCR4 was required for MSC migration [35]. The SDF-1/CXCR4 axis is critical for MSC migration [36]; an SDF-1 pretreatment, acting through its receptor CXCR4 on MSCs, significantly activated the PI3K/Akt and ERK1/2 signalling pathways in MSCs, and these effects were partially inhibited by AMD3100 [36]. Therefore, in our study, we further explored whether the ERK1/2 and PI3K/Akt signalling pathways are involved in the migration and homing of hAD-MSCs mediated by the SDF-1/CXCR4 axis.

In this study, the role of the SDF-1/CXCR4 axis in the migration and homing of systemically transplanted hAD-MSCs to the ovaries of rats with chemotherapy-induced POI and its relevant downstream signalling pathways were explored. These findings may provide new evidence to improve our understanding of the molecular mechanisms involved in the migration and homing of hAD-MSCs to ovaries exhibiting POI.

## Methods

The experimental protocols were performed in compliance with the Declaration of Helsinki and approved by the Ethics Committee of the Second Affiliated Hospital of Chongqing Medical University.

### Reagents

Low-glucose Dulbecco’s modified Eagle’s medium (L-DMEM) and foetal bovine serum (FBS) were purchased from Gibco Co. (Grand Island, NY, USA). Cell Counting Kit-8 (CCK-8), penicillin, streptomycin, TUNEL apoptosis assay kit, Bradford Protein Assay Kit, RIPA lysis buffer and BeyoECL Plus kit were purchased from Beyotime Institute of Biotechnology (Haimen, China). Adipogenic differentiation medium (ADM), osteogenic differentiation medium (ODM), chondrogenic differentiation medium (CDM), Oil Red O, Alcian blue and Alizarin Red S were purchased from Cyagen Biosciences Inc. (Suzhou, China). 2-(4-Amidinophenyl)-6-indolecarbamidine dihydrochloride (DAPI) and phosphate-buffered saline (PBS) were purchased from Boster Biological Technology Co., Ltd. (Wuhan, Hubei, China). The CXCR4 antibody was purchased from Novus Biologicals (Littleton, CO, USA). Recombinant human SDF-1 was purchased from PeproTech Inc. (Cranbury, NJ, USA). SDF-1, anti-Müllerian hormone (AMH), oestradiol (E2) and follicle-stimulating hormone (FSH) ELISA kits were purchased from Uscn Life Science Inc. (Wuhan, Hubei, China). The Bax antibody, LY294002 and secondary antibodies were purchased from Cell Signaling Technology Inc. (Boston, MA, USA). DyLight549-conjugated antibodies were purchased from Abbkine Scientific Co., Ltd. (Liyang, Jiangsu, China). AMD3100 was purchased from MedChemExpress (Monmouth Junction, NJ, USA). Cleaved caspase 3, Bcl-2, vascular endothelial growth factor (VEGF) and vascular endothelial growth factor receptor 2 (VEGFR2) antibodies were purchased from Affinity Biosciences (Wuhan, Hubei, China). Cyclophosphamide was purchased from Hengrui Medicine Co., Ltd. (Lianyungang, Jiangsu, China). All other chemicals were purchased from Sigma–Aldrich Co. (St. Louis, MO, USA).

### Isolation and culture of hAD-MSCs

Primary hAD-MSCs were isolated from term amnions as described in our previous protocols [9]. Term placentas were collected from healthy donors who received caesarean section at the Second Affiliated Hospital of Chongqing Medical University, Chongqing, China. Written informed consent was obtained from all donors before tissue collection. hAD-MSCs were cultured in L-DMEM supplemented with 12% FBS, 100 U/mL penicillin and 0.1 mg/mL streptomycin. hAD-MSCs were used at the third passage in the subsequent experiments.

### Identification and characterization of hAD-MSCs

hAD-MSCs were identified using our previously published protocols [9]. The morphological characteristics and growth of hAD-MSCs were observed and imaged with an inverted microscope (Olympus Corporation, Tokyo, Japan). The expression of MSC surface markers on hAD-MSCs was detected using flow cytometry. To identify the multipotent differentiation of hAD-MSCs, hAD-MSCs were cultured in ADM, ODM and CDM for 21 days to identify the multidifferentiation potential of hAD-MSCs. After staining with Oil Red O, Alizarin Red S or Alcian blue, the cells were observed and imaged under an inverted microscope (Olympus Corporation, Tokyo, Japan).

The growth curve of hAD-MSCs was constructed by performing a CCK-8 assay according to the manufacturer’s instructions. Cells were seeded at a density of 5 × 10^3^ cells/well in 96-well plates and cultured for 24 h. Then, the optical density (OD) value at 450 nm was measured daily for 5 continuous days using a plate reader (model 1510; Thermo Fisher Scientific Oy, Vantaa, Finland).

### Transwell migration assay

An in vitro cell migration model was constructed in a Transwell chamber (Corning, NY, USA) composed of a membrane filter with 8 μm diameter pores suspended in a 6-well plate according to the manufacturer’s instructions. hAD-MSCs were plated in serum-free medium at a density of 1 × 10^5^/cm^2^ into the upper chamber, and the lower chamber was filled with the same medium containing 2% FBS. For inhibitor treatment, hAD-MSCs were preincubated with AMD3100 (44 nM, a specific inhibitor of CXCR4) or LY294002 (50 μmol/L, a specific inhibitor of the PI3K/Akt pathway) for 1 h. For SDF-1 treatment, SDF-1 was added at concentrations of 0, 50, 100 or 150 ng/mL to the lower chamber according to previously published protocols [24, 25, 37]. After 24 h, migration assays were terminated by retrieving the membrane filter from each group, and hAD-MSCs on the underside of the filter were stained with a crystal violet staining solution and counted in 6 randomly chosen visual fields (200×) under a microscope (Olympus Corporation, Tokyo, Japan).

### Western blot

hAD-MSCs were collected from four different persons to examine CXCR4 expression in hAD-MSCs. Serum-starved hAD-MSCs were pretreated with AMD3100 (44 nM) or LY294002 (50 μmol/L) for 1 h followed by treatment with SDF-1 (100 ng/mL) to explore the mechanisms associated with hAD-MSC migration mediated by the SDF-1/CXCR4 axis. After treatment, hAD-MSCs were lysed in RIPA lysis buffer, and proteins were isolated after centrifugation. The protein concentrations were determined using the Bradford Protein Assay Kit. The protein samples were separated on SDS–PAGE gels and subsequently electrotransferred to PVDF membranes (Millipore, USA). After washing, the membranes were blocked with 5% skim milk for 1 h and incubated with specific primary antibodies against CXCR4, Akt, phospho-Akt (Ser473), ERK1/2 or phospho-ERK1/2 overnight at 4 °C. After washing, the membranes were incubated with secondary antibodies for 1 h at room temperature, and a BeyoECL Plus kit was used for colour development according to the manufacturer’s instructions.

### Immunofluorescence staining

Cells were detected using immunofluorescence staining to further confirm CXCR4 expression in hAD-MSCs. The cells were fixed, washed and permeabilized in PBS containing 0.5% Triton X-100 for 30 min. After washing, the cells were blocked with 5% BSA for 2 h and incubated with a specific primary antibody against CXCR4 overnight at 4 °C. After washing, the cells were incubated with secondary antibodies conjugated to DyLight549 for 1 h at 37 °C. Then, the cells were counterstained with DAPI and imaged under a laser scanning confocal microscope (Nikon Corporation, Tokyo, Japan).

### Labelling and tracking of hAD-MSCs

Cells were prelabelled with PKH26 Red Fluorescent Cell Linker Kits using our previously published protocols [8] before transplantation to track and locate the transplanted hAD-MSCs in the ovarian tissue. At 24 h after the transplantation of PKH26-labelled hAD-MSCs, fresh sections of ovaries were prepared and incubated with DAPI, and the sections were imaged under a laser scanning confocal microscope (Nikon Corporation, Tokyo, Japan).

The homing efficiency of hAD-MSCs within ovarian tissue is typically quantified by averaging the number of PKH26-labelled cells present in 800 × microscopic fields randomly chosen from each tissue sample under a confocal microscope using a published protocol [16].

### Animal protocols

Female Sprague–Dawley (SD) rats aged 10‒12 weeks were purchased from the Experimental Animal Center of Chongqing Medical University.

Seventy-two female SD rats were randomly divided into the following 4 groups to investigate whether the SDF-1/CXCR4 axis mediates the homing of hAD-MSCs to the ovaries of rats with chemotherapy-induced POI: control, POI, hAD-MSCs and hAD-MSCs + AMD3100 groups (*n* = 18 rats in each group). First, the rats were intraperitoneally injected with cyclophosphamide to establish POI models in the POI, hAD-MSCs and hAD-MSCs + AMD3100 groups using our previously published protocols [8]. For the inhibitor treatment, hAD-MSCs were incubated with AMD3100 (44 nM) for 1 h before cell transplantation into the hAD-MSCs + AMD3100 group. Then, at 24 h after chemotherapy, rats from the hAD-MSCs and hAD-MSCs + AMD3100 groups were injected with 0.6 ml of PBS containing 4 × 10^6^ hAD-MSCs labelled with PKH-26 via the tail vein, while the rats in the control and POI groups were injected with 0.6 ml of PBS, according to our previously published protocols [8, 11]. At 24 h, 3 weeks and 6 weeks after cell transplantation, 6 rats from each group were sacrificed under sodium pentobarbital anaesthesia, and samples were collected for the subsequent experiments.

The oestrous cycles of rats in each group were recorded by observing vaginal smears, as described in our previously published protocols [8]. Regular oestrous cycles consisted of 4 sequential stages as follows: proestrus, oestrus, metoestrus and dioestrus (Fig. 6a). Irregular oestrous cycles were also defined in our previously published protocols [8].

Twenty-four female SD rats were randomly divided into the following 4 groups to further investigate the molecular mechanisms involved in the homing of hAD-MSCs mediated by the SDF-1/CXCR4 axis in vivo: control, POI, hAD-MSCs and hAD-MSCs + LY294002 groups (*n* = 6 rats in each group). Rats were intraperitoneally injected with cyclophosphamide to establish POI models in the POI, hAD-MSCs and hAD-MSCs + LY294002 groups. For the inhibitor treatment, hAD-MSCs were incubated with LY294002 (50 μmol/L) for 1 h before cell transplantation into the hAD-MSCs + LY294002 group. At 24 h after chemotherapy, rats from the hAD-MSCs and hAD-MSCs + LY294002 groups were injected with 0.6 ml of PBS containing 4 × 10^6^ hAD-MSCs labelled with PKH-26 via the tail vein, while rats in the control and POI groups were injected with 0.6 ml of PBS. At 24 h after cell transplantation, rats in each group were sacrificed under sodium pentobarbital anaesthesia, and samples were collected for tracking tests.

### Enzyme-linked immunosorbent assay (ELISA)

Ovarian tissue and serum were collected from the rats in the control and POI groups to detect the SDF-1 levels in the ovaries and serum of rats with POI at 24 h after chemotherapy. Ovarian tissue was homogenized, and the supernatant was collected. The SDF-1 levels were detected using an ELISA kit according to the manufacturer’s instructions.

Serum was collected at 0, 3 and 6 weeks after cell transplantation and levels of AMH, FSH and E2 in rats from each group were analysed using ELISA kits according to the manufacturer’s instructions.

### Analysis of ovarian morphology and follicle counts

Ten ovaries from each group were collected at 6 weeks after hAD-MSC transplantation. Ovaries were fixed, dehydrated, embedded in paraffin and cut into 5 μm sections. The sections were stained with haematoxylin and eosin (HE). The ovarian morphology was observed under an optical microscope (Olympus Corporation, Tokyo, Japan). The follicles in ovaries were classified as primordial, primary, secondary, preovulatory and atretic follicles. The number of follicles was counted as described in previous studies [3, 11].

### TUNEL assay

Ovarian cell apoptosis was determined using a TUNEL apoptosis assay kit according to the manufacturer’s instructions. Sections were observed and imaged with an optical microscope (Olympus Corporation, Tokyo, Japan). Nuclei of ovarian apoptotic cells were stained dark brown.

### Immunohistochemical staining

Ovarian tissue sections were incubated with specific primary antibodies against Bcl-2, Bax, cleaved caspase-3, VEGF and VEGFR2, followed by the corresponding secondary antibodies conjugated to horseradish peroxidase. Afterwards, sections were stained with 3,3′-diaminobenzidine and counterstained with haematoxylin. Then, the sections were observed and imaged under an optical microscope (Olympus Corporation, Tokyo, Japan). The sections were analysed as described in our previously published protocols [8]: the number of positive cells was graded as 4 (> 75%), 3 (51‒75%), 2 (25‒50%), 1 (5‒25%) or 0 (< 5%), and the staining intensity was graded as 3 (brown), 2 (light brown), 1 (light yellow) or 0 (no colour). The total score was calculated as the sum of the two grades, which was named the immunoreactivity score (IS). Ten high-power fields (HPFs, 400 ×) were randomly chosen from five sections in each group for scoring. The median and range of ISs for each group were calculated.

### Statistical analysis

For all assays, at least three independent experiments were performed. Statistical analyses of data were processed using SPSS 22.0 software (IBM, NY, USA). Data with a normal distribution are presented as the means ± standard deviations, and an independent samples *t-*test and one-way analysis of variance (ANOVA) were used for two-group and multiple-group comparisons, respectively. Data with a skewed distribution are presented as the medians and ranges, and the nonparametric Wilcoxon rank test and Kruskal–Wallis test were used for two-group and multiple-group comparisons, respectively. Statistical significance was set to *P* < 0.05.

## Results

### Characterization and identification of hAD-MSCs

Primary hAD-MSCs were isolated, cultured and identified as described in our previously published protocols [9]. hAD-MSCs were identified according to the guidelines established by the International Society of Cell Therapy (ISCT).

**Fig. 1:**
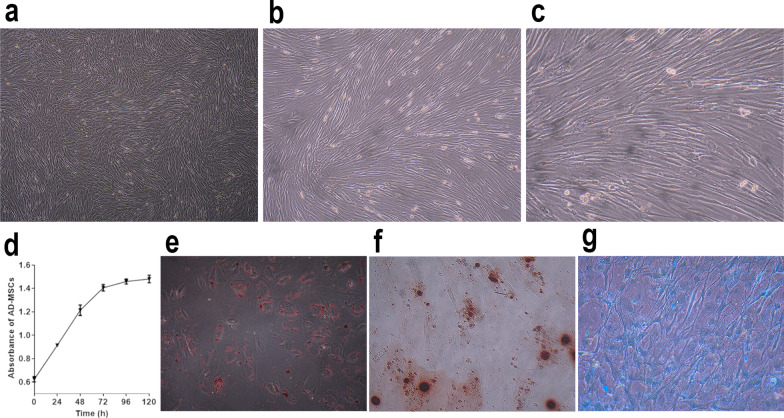
Characterization of hAD-MSCs. **a–c** Morphology of hAD-MSCs (**a** ×40, **b** ×100, **c** ×200). **d** The growth curve of hAD-MSCs. **e–g** hAD-MSCs were able to differentiate into adipocytes (**e** ×200), osteoblasts (**f** ×200) and chondroblasts (**g** ×200). Lipid vacuoles in the cytoplasm were visualized in adipocytes, which were verified by Oil Red O staining (**e**). Abundant calcium deposits were visualized in osteoblasts, which were stained with Alizarin Red S (**f**). Cartilage-specific proteoglycans (**g**) were visualized in chondroblasts stained with Alcian blue. Representative images are shown

The isolated cells grew as adherent cultures, displayed a fibroblastic morphology and were capable of forming colonies (Fig. 1a-c). The growth curve of hAD-MSCs was investigated by performing a CCK-8 assay (Fig. 1d). The expression of surface markers on hAD-MSCs was similar to that on BM-MSCs, which was detected and published in our previous study [9]. The isolated cells were confirmed to have the ability to differentiate into adipocytes, osteoblasts and chondroblasts under standard differentiation conditions in vitro, which were verified by Oil Red O, Alizarin Red S and Alcian blue staining (Fig. 1e-g).

Based on these results, the isolated cells have the common characteristics of MSCs and are identified as hAD-MSCs.

### Expression of CXCR4 in hAD-MSCs

**Fig. 2:**
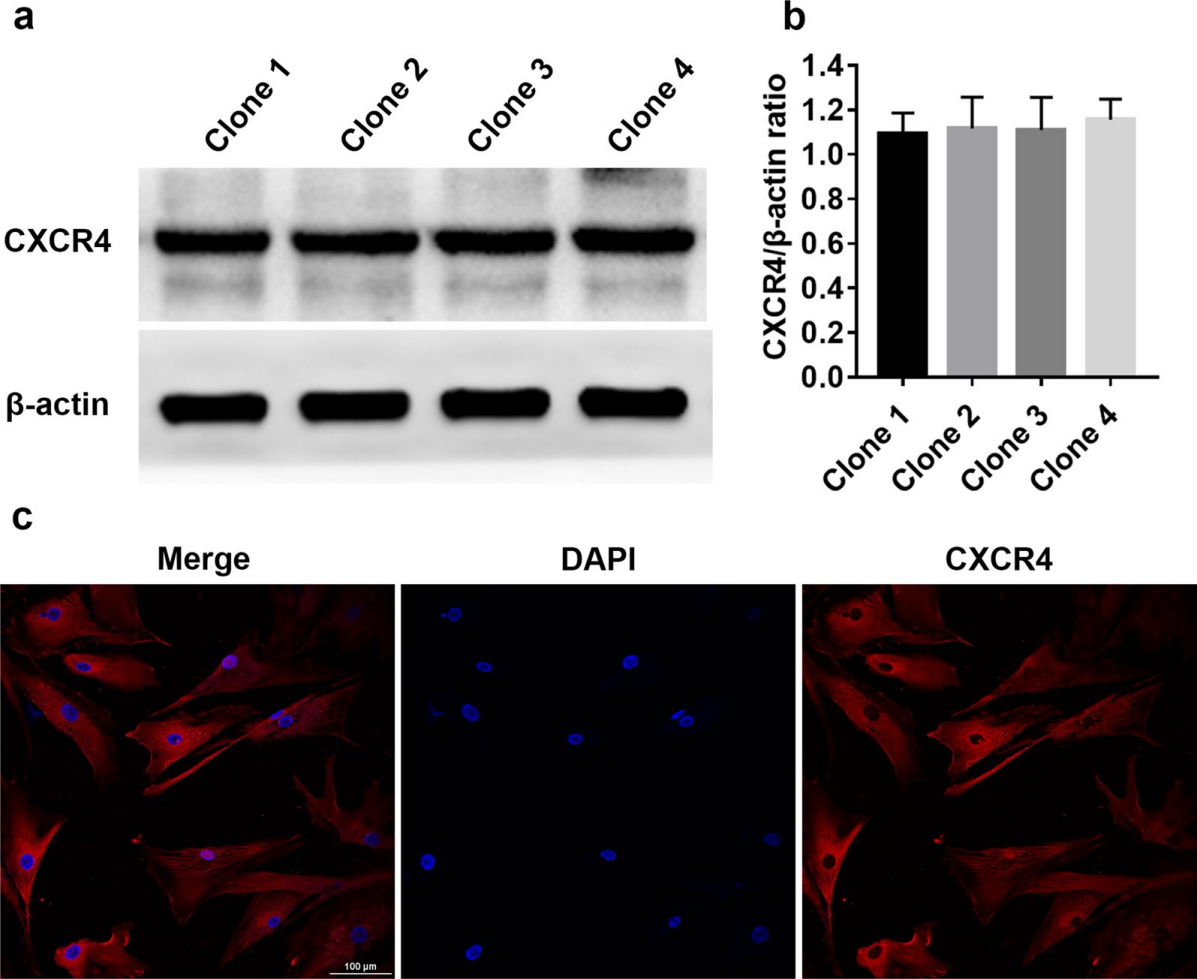
CXCR4 expression in hAD-MSCs. **a**, **b** Western blot assay was performed to detect CXCR4 protein expression levels in hAD-MSCs (**a**), and the CXCR4/β-actin ratios were evaluated (**b**). **c** Immunofluorescence staining was performed to confirm the expression of the CXCR4 protein in hAD-MSCs (×200). Representative images are shown. *P < 0.05 and **P < 0.01. Scale bars = 100 μm

The expression of CXCR4 in hAD-MSCs was examined by performing Western blotting and immunofluorescence staining. CXCR4 was stably expressed in hAD-MSCs, and no significant difference was observed in different cell clones (*P* > 0.05; Fig. 2a, b). Red fluorescent signals, which indicated CXCR4 expression, were observed in hAD-MSCs (Fig. 2c).

### SDF-1 induces the migration of hAD-MSCs in vitro

**Fig. 3:**
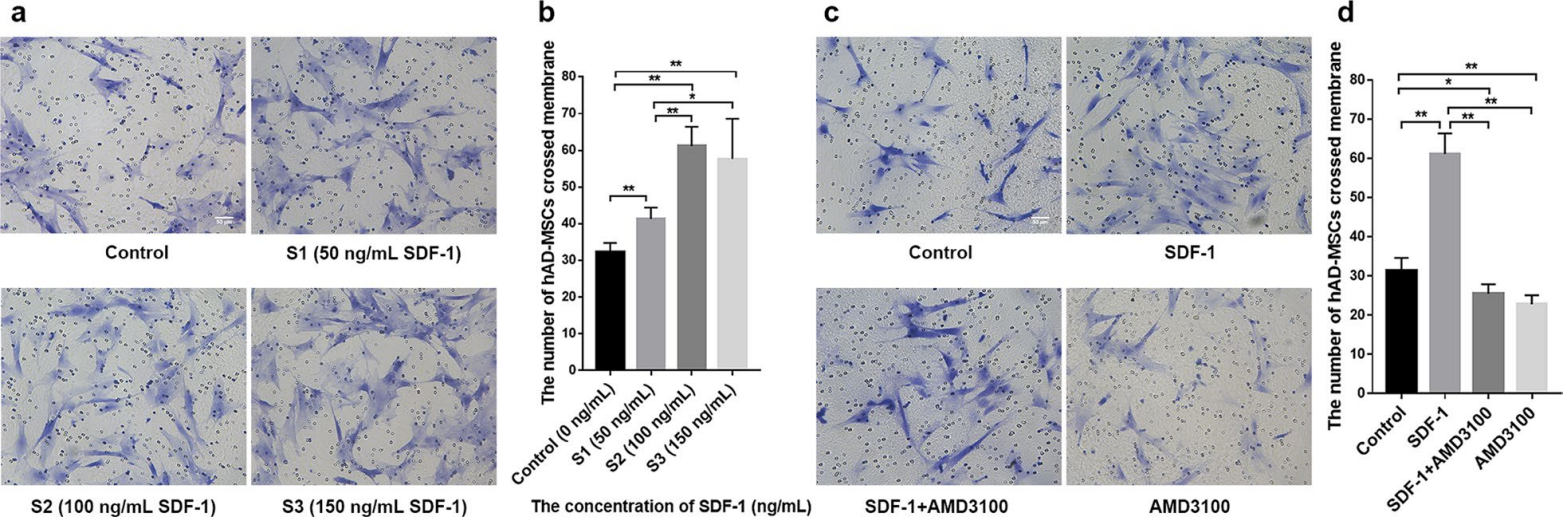
SDF-1 induces hAD-MSC migration in vitro. **a**, **b** The migration of hAD-MSCs was tested by performing a Transwell migration assay with 0, 50, 100 or 150 ng/mL SDF-1 in vitro (×200). **c**, **d** The role of the SDF-1/CXCR4 axis in the SDF-1-induced migration of hAD-MSCs was detected by performing a Transwell migration assay. The migration of hAD-MSCs was tested in the control, SDF-1, SDF-1 + AMD3100 and AMD3100 groups (×200). Representative images are shown. *N* = 6, *P < 0.05 and **P < 0.01. Scale bars = 50 μm

A Transwell migration assay was performed with 0, 50, 100 or 150 ng/mL SDF-1 to explore whether SDF-1 can promote hAD-MSC migration in vitro and determine the optimal dose of SDF-1 for subsequent experiments. Compared to the control group (0 ng/mL SDF-1), the number of hAD-MSCs passing through the membrane in wells containing SDF-1 was significantly greater in the S1 (50 ng/mL SDF-1), S2 (100 ng/mL SDF-1) and S3 (150 ng/mL SDF-1) groups (*P* < 0.01; Fig. 3a, b). Compared to the S1 group, the number of hAD-MSCs passing through the membrane in response to SDF-1 was significantly greater in the S2 and S3 groups (*P* < 0.01 and *P* < 0.05; Fig. 3a, b). However, no significant difference was observed between the S2 and S3 groups (*P* > 0.05; Fig. 3a, b). Thus, an SDF-1 concentration of 100 ng/ml was selected for the subsequent experiments.

AMD3100, a specific inhibitor of CXCR4, was used in the Transwell migration assay to further explore the role of the SDF-1/CXCR4 axis in the SDF-1-induced migration of hAD-MSCs in vitro. Compared to the control group, the number of hAD-MSCs passing through the membrane was significantly greater in the SDF-1 group (*P* < 0.01; Fig. 3c, d). However, the increased number of hAD-MSCs passing through the membrane in response to SDF-1 was significantly inhibited by AMD3100 (*P* < 0.01; Fig. 3c, d). Compared to the SDF-1 group, the number of hAD-MSCs passing through the membrane was significantly decreased in the SDF-1 + AMD3100 group (*P* < 0.01; Fig. 3c, d).

Thus, SDF-1 induces the migration of hAD-MSCs in vitro, and the SDF-1/CXCR4 axis mediates the SDF-1-induced migration of hAD-MSCs.

### Increased SDF-1 levels in serum and ovarian tissue from rats with chemotherapy-induced POI

**Fig. 4:**
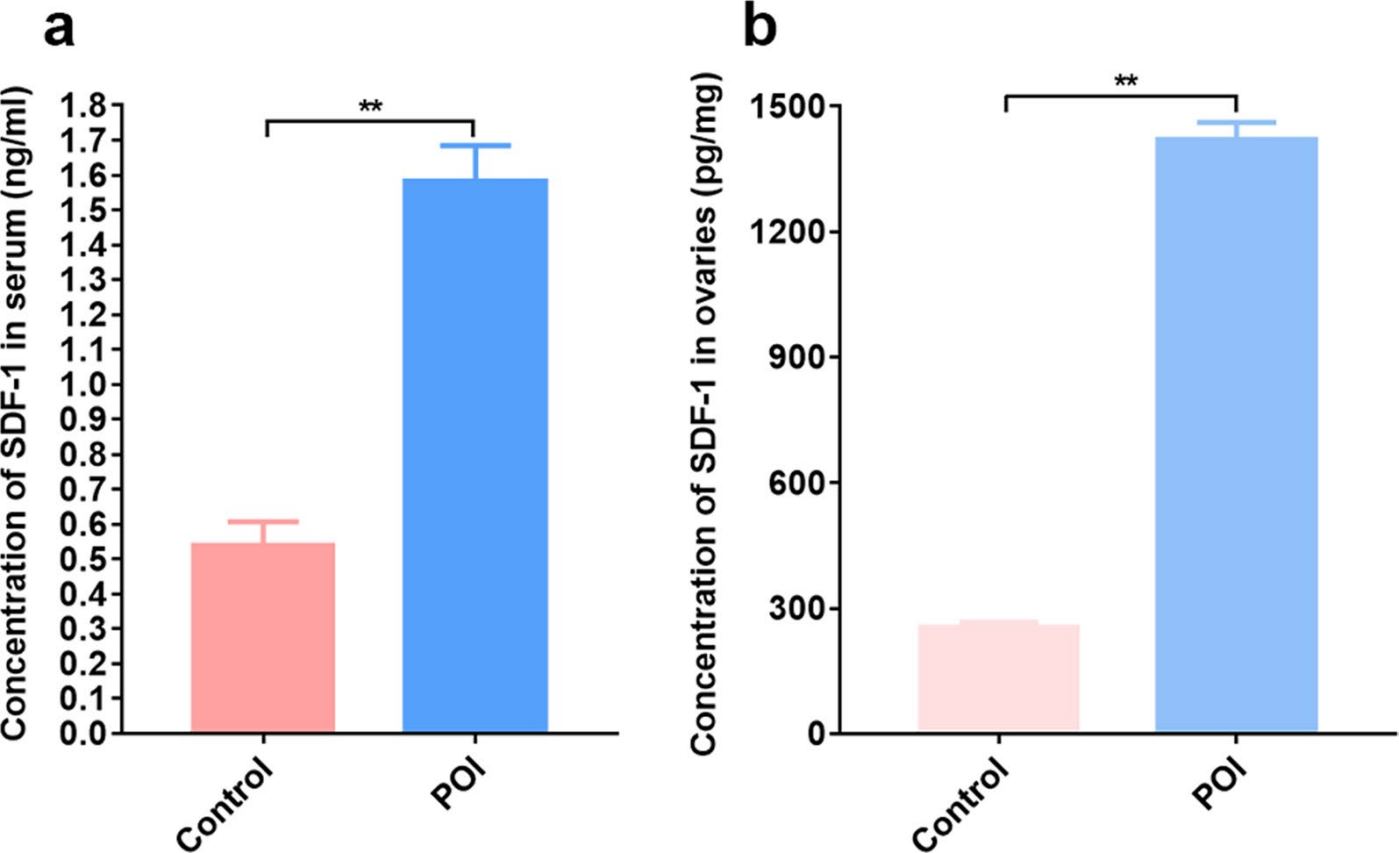
Elevated levels of SDF-1 in serum and ovaries are induced by chemotherapy in rats with POI. SDF-1 concentrations in the serum (**a**) and supernatant of homogenized ovarian tissue (**b**) from the control and POI groups were measured using ELISA. *P < 0.05 and **P < 0.01

Levels of SDF-1 in the serum and ovaries in the control and POI groups were measured after chemotherapy to examine whether chemotherapy induces SDF-1 secretion in the rat ovaries. Compared to the control group, SDF-1 levels in the serum and ovaries were significantly increased in the POI group (*P* < 0.01; Fig. 4a, b).

The results indicate that chemotherapy induces SDF-1 secretion in ovaries and subsequently increases serum SDF-1 levels in rats. The injured ovaries in rats with chemotherapy-induced POI might attract hAD-MSCs expressing CXCR4.

### The SDF-1/CXCR4 axis partially mediates the homing of hAD-MSCs to the ovaries of rats with chemotherapy-induced POI

**Fig. 5:**
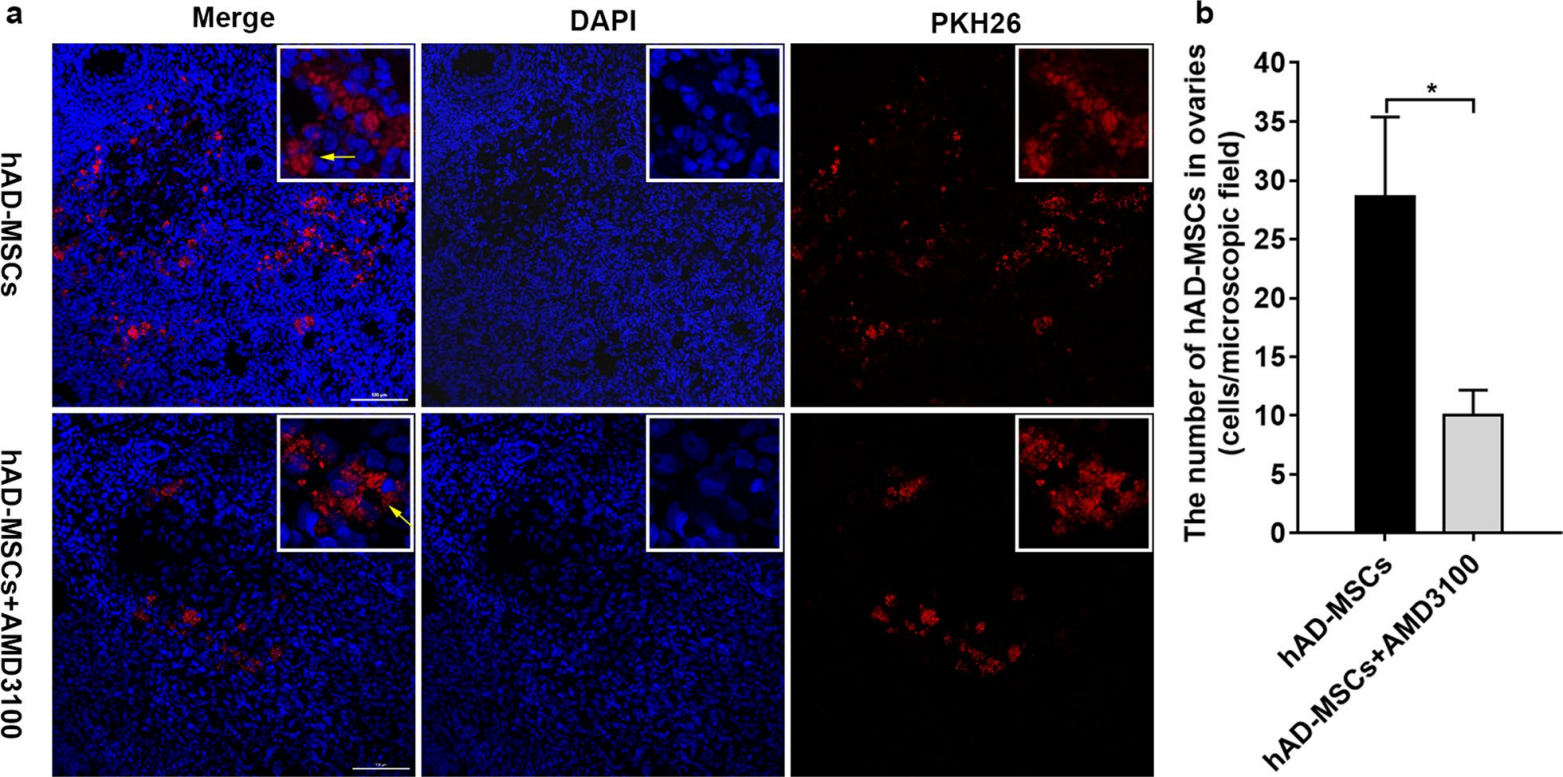
The SDF-1/CXCR4 axis is involved in the homing of hAD-MSCs to the ovaries of rats with chemotherapy-induced POI. **a** Transplanted PKH26-labelled hAD-MSCs were observed in ovaries at 24 h after cell transplantation in the hAD-MSCs and hAD-MSCs + AMD3100 groups using confocal microscopy (×200 and ×800). **b** The number of hAD-MSCs was counted and compared in the hAD-MSCs and hAD-MSCs + AMD3100 groups (*n* = 10). Representative images are shown. The yellow arrows indicate transplanted hAD-MSCs. *P < 0.05. Scale bars = 100 μm

hAD-MSCs were prelabelled with PKH26 before transplantation using our previously published protocols to track and locate the transplanted hAD-MSCs in vivo [8], and PKH26-labelled hAD-MSCs showed red fluorescence. The homing and location of transplanted PKH26-labelled hAD-MSCs in the ovaries of rats with POI were traced at 24 h after cell transplantation. PKH26-labelled cells, which showed red-dotted fluorescent signals, were recruited and located in the interstitium of ovaries from rats with POI in the hAD-MSCs and hAD-MSCs + AMD3100 groups after transplantation (Fig. 5a). No punctate red fluorescent signals were observed in the control and POI groups, consistent with our previously published articles [8, 11] (data not shown). Quantitation of the number of hAD-MSCs in ovaries revealed that ovaries from rats in the hAD-MSCs and hAD-MSCs + AMD3100 groups contained 28.60 ± 21.49 and 10.00 ± 6.85 cells/microscopic field, respectively (*n* = 10; Fig. 5b). The intensity of punctate red fluorescent signals and number of hAD-MSCs in the hAD-MSCs + AMD3100 group were significantly lower than those of hAD-MSCs in the hAD-MSCs group (*P* < 0.05; Fig. 5a, b).

Based on these results, the ovaries of rats with chemotherapy-induced POI attract hAD-MSCs expressing CXCR4 in vivo. The SDF-1/CXCR4 axis partially mediates the homing of systemically transplanted hAD-MSCs to the ovaries of rats with chemotherapy-induced POI.

### Effects of hAD-MSCs on ovarian function in rats with chemotherapy-induced POI after blocking the SDF-1/CXCR4 axis

Oestrous cycles and sex hormone levels in rats were analysed to investigate the effects of hAD-MSC transplantation on ovarian function in rats with chemotherapy-induced POI after blocking the SDF-1/CXCR4 axis.

**Fig. 6:**
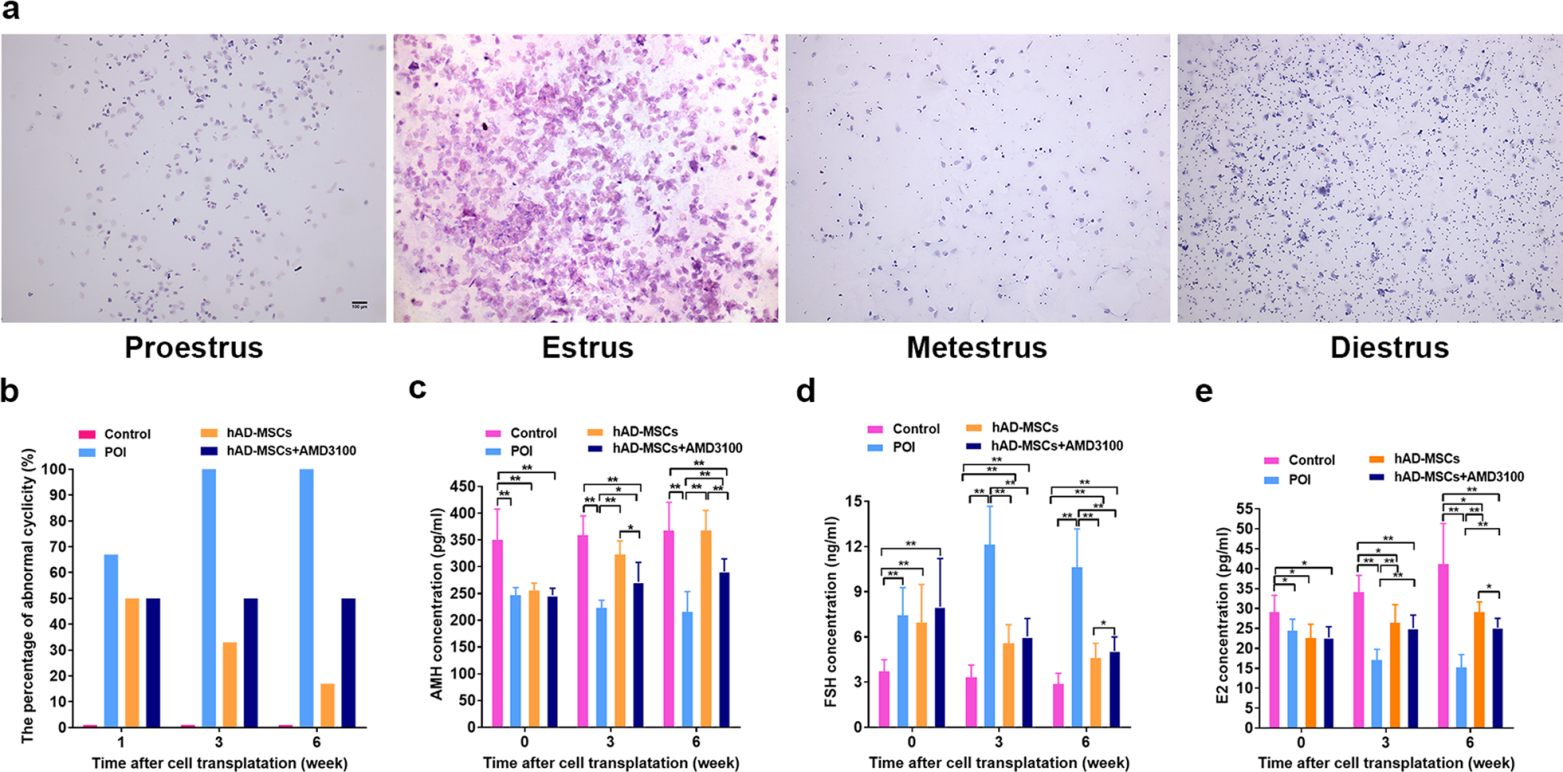
Effects of hAD-MSCs on ovarian function in rats with chemotherapy-induced POI after blocking the SDF-1/CXCR4 axis. **a** Oestrous cycles of rats were observed. Regular oestrous cycles consisted of 4 sequential stages: proestrus, oestrus, metoestrus and dioestrus (×40). **b** The percentages of rats with abnormal cyclicity were detected at 1, 3, and 6 weeks after cell transplantation. **c–e** Serum levels of AMH (**c**), FSH (**d**) and E2 (**e**) were detected at 0, 3 and 6 weeks after cell transplantation. Representative images are shown. *P < 0.05 and **P < 0.01. Scale bars = 100 μm

Beginning at the 3rd week after hAD-MSC transplantation, 100% of the rats in the POI group had irregular oestrous cycles, while 100% of the rats in the control group had regular oestrous cycles (Fig. 6a, b). Beginning at the 3rd week after hAD-MSC transplantation, the percentages of rats with irregular oestrous cycles were lower in the hAD-MSCs and hAD-MSCs + AMD3100 groups than in the POI group (Fig. 6b), while greater percentages of rats with irregular oestrous cycles were observed in the hAD-MSCs + AMD3100 group than in the hAD-MSCs group (Fig. 6b).

After chemotherapy, the FSH level was significantly higher (*P* < 0.05; Fig. 6d) and the AMH and E2 levels were significantly lower (*P* < 0.05; Fig. 6c, e) in the POI, hAD-MSCs and hAD-MSCs + AMD3100 groups than in the control group, and no significant differences were between the first three groups (*P* > 0.05; Fig. 6c–e). Beginning at the 3rd week after hAD-MSC transplantation, the FSH level was significantly lower (*P* < 0.05; Fig. 6d), while the AMH and E2 levels were significantly higher (*P* < 0.05; Fig. 6c, e) in the hAD-MSCs and hAD-MSCs + AMD3100 groups than in the POI group. Moreover, compared to the hAD-MSCs group, a significantly lower AMH level was detected in the hAD-MSCs + AMD3100 group from the 3rd week after hAD-MSC transplantation (*P* < 0.05; Fig. 6c). Compared to the hAD-MSCs group, the FSH level was significantly higher (*P* < 0.05; Fig. 6d) and the E2 level was significantly lower (*P* < 0.05; Fig. 6e) in the hAD-MSCs + AMD3100 group at the 6th week after hAD-MSC transplantation.

Therefore, hAD-MSC transplantation improves ovarian function in rats with chemotherapy-induced POI, and blocking the SDF-1/CXCR4 axis with a CXCR4 antagonist reduces its efficacy in POI treatment.

### Effects of hAD-MSCs on ovarian injuries induced by chemotherapy in rats with POI after blocking the SDF-1/CXCR4 axis

Ovaries were collected for a pathological analysis at 6 weeks after cell transplantation to investigate the effects of hAD-MSC transplantation on ovarian injuries in rats with chemotherapy-induced POI after blocking the SDF-1/CXCR4 axis.

**Fig. 7:**
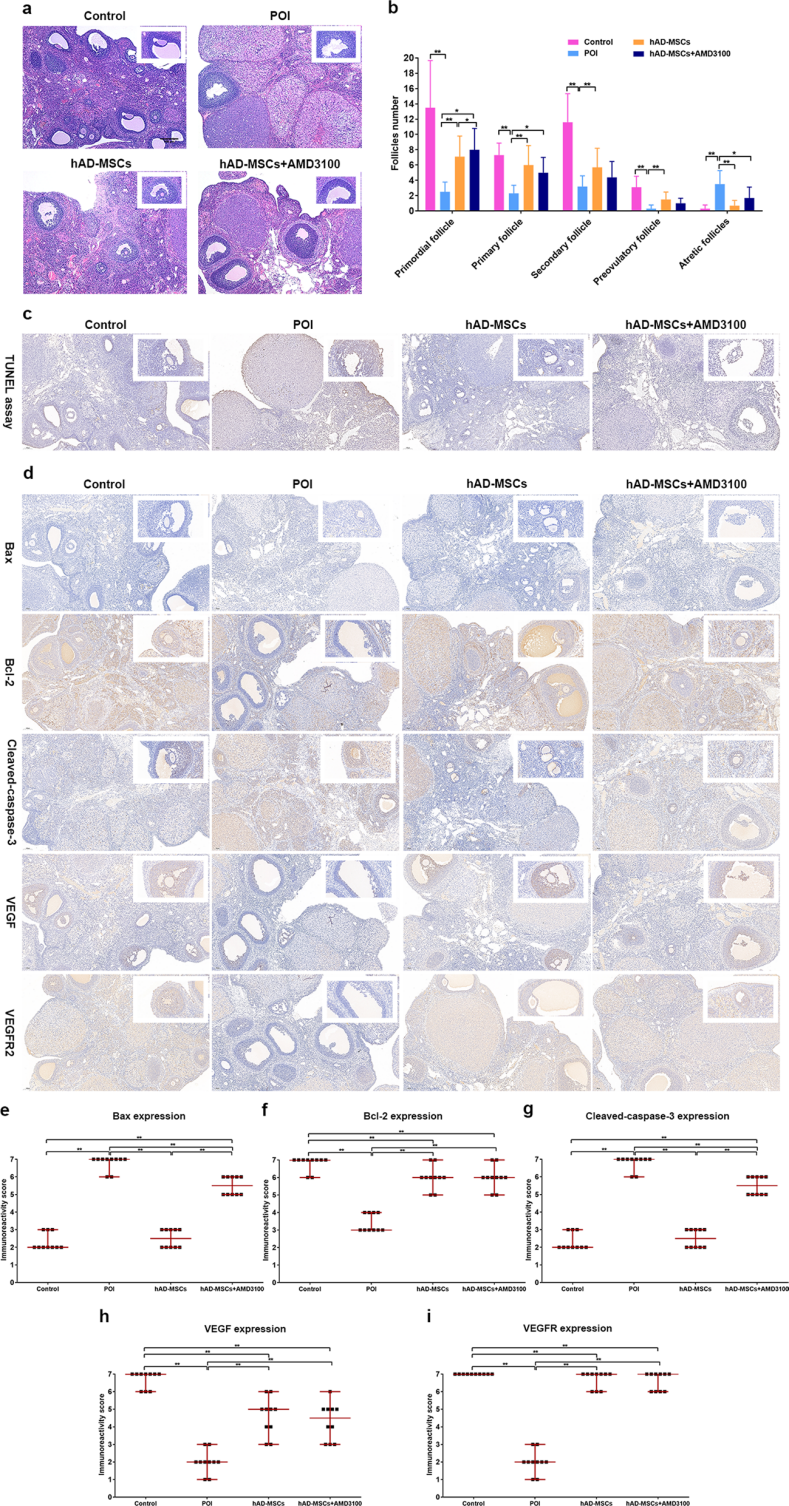
Effects of hAD-MSCs on ovarian injuries induced by chemotherapy in POI rats after blocking the SDF-1/CXCR4 axis. **a** Changes in the ovarian tissue were analysed using HE staining (×100 and ×200). **b** The number of follicles at different stages was counted and compared in the control, POI, hAD-MSCs and hAD-MSCs + AMD3100 groups (*n* = 10). **c** Ovarian granulosa cell (GC) apoptosis was tested using the TUNEL assay (×100 and ×400). **d** The expression levels of Bax, Bcl-2, cleaved-caspase-3, VEGF and VEGFR2 in the ovaries were detected using immunohistochemical staining (×100 and ×400). **e–i** Semiquantitative analyses of Bax, Bcl-2, cleaved-caspase-3, VEGF and VEGFR2 levels in the ovaries from each group are shown (*n* = 10). Each dot in the graphs (**e–i**) represents the value obtained from ten high-power fields (HPFs) randomly chosen from five sections in each group. The bars and error bars in graphs (**e–i**) indicate the medians and ranges, respectively. Brown cells represent immunostained cells. Representative images are shown. *P < 0.05 and **P < 0.01. Scale bars = 100 μm

Compared to the control group, chemotherapy induced follicle loss, vascular damage, and tissue fibrosis in ovaries from the POI group (Fig. 7a). Meanwhile, compared to the POI group, hAD-MSC transplantation reduced follicle loss, vascular damage, and tissue fibrosis in the hAD-MSCs and hAD-MSCs + AMD3100 groups (Fig. 7a). Compared to the control group, the numbers of primordial follicles and growing follicles, including primary, secondary, and preovulatory follicles, were significantly lower in the POI group (*P* < 0.05; Fig. 7a, b). Compared to the POI group, significantly greater numbers of primordial and primary follicles were observed in the hAD-MSCs and hAD-MSCs + AMD3100 groups (*P* < 0.05; Fig. 7a, b). Moreover, compared to the hAD-MSCs group, a significantly lower number of primordial follicles was observed in the hAD-MSCs + AMD3100 group (*P* < 0.05; Fig. 7a, b).

Compared to the control group, a significantly greater number of apoptotic granulosa cells (GCs) in the ovaries was observed in the POI group (Fig. 7c). Compared to the POI group, the number of apoptotic GCs in ovaries was significantly lower in the hAD-MSCs and hAD-MSCs + AMD3100 groups (Fig. 7c). Moreover, compared to the hAD-MSCs group, the number of apoptotic GCs in ovaries was significantly higher in the hAD-MSCs + AMD3100 group (Fig. 7c).

Compared to the control group, significantly higher levels of Bax and cleaved caspase-3 (proapoptotic protein) (*P* < 0.01; Fig. 7d–i) and significantly lower levels of Bcl-2 (antiapoptotic protein), VEGF and VEGFR2 (*P* < 0.01; Fig. 7d–i) were detected in the POI group. Significantly lower levels of Bax and cleaved caspase-3 (*P* < 0.01; Fig. 7d–i) and significantly higher levels of Bcl-2, VEGF and VEGFR2 were detected (*P* < 0.01; Fig. 7d–i) in the hAD-MSCs and hAD-MSCs + AMD3100 groups than in the POI group. Moreover, compared to the hAD-MSCs group, the levels of Bax and cleaved caspase-3 were significantly higher in the hAD-MSCs + AMD3100 group (*P* < 0.01; Fig. 7d–i).

These results reveal that hAD-MSC transplantation reduces ovarian injuries, inhibits GC apoptosis and proapoptotic protein expression, and promotes antiapoptotic protein, VEGF and VEGFR2 expression in the ovaries of rats with POI. Blocking the SDF-1/CXCR4 axis with a CXCR4 antagonist reduces the efficacy of hAD-MSC transplantation.

### Activation of the PI3K/Akt signalling pathway by SDF-1 in hAD-MSCs

The PI3K/Akt and ERK1/2 signalling pathways were detected to explore the mechanisms by which SDF-1/CXCR4 chemotactic homing signals are transmitted within hAD-MSCs.

**Fig. 8:**
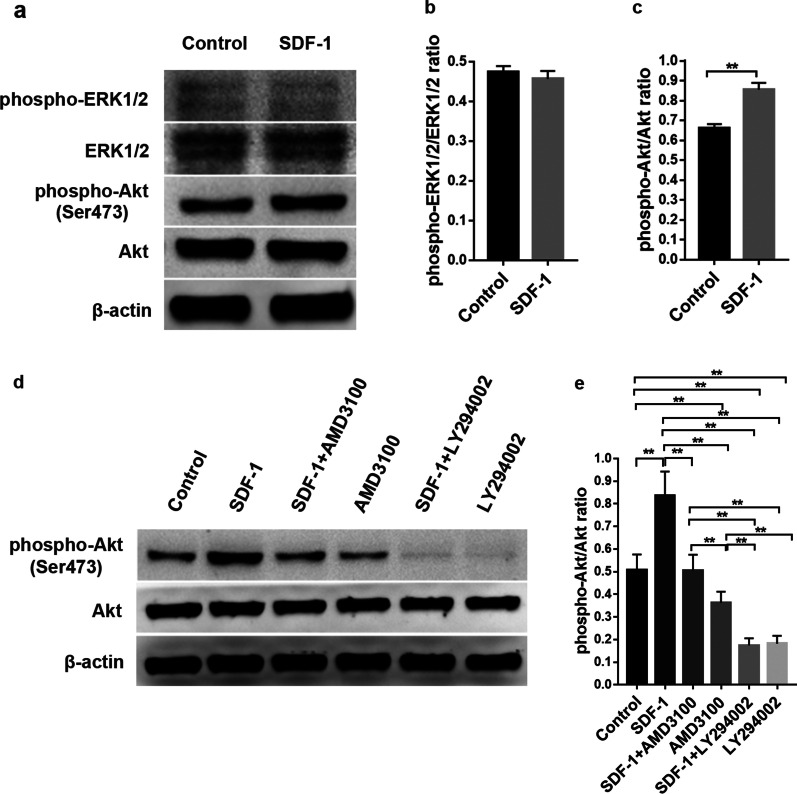
Activation of the PI3K/Akt signalling pathway by SDF-1 in hAD-MSCs. **a–c** The effects of SDF-1 on the phosphorylation of ERK1/2 and Akt in hAD-MSCs were determined using Western blot analysis (**a**), and the phospho-ERK1/2/ERK1/2 and phospho-Akt/Akt ratios were evaluated (**b**, **c**). **d**, **e** Akt and phospho-Akt levels were analysed using Western blotting after pretreatment of hAD-MSCs with AMD3100 or LY294002 for 1 h followed by treatment with SDF-1 (**d**), and the phospho-Akt/Akt ratios were evaluated (**e**). *P < 0.05 and **P < 0.01

Following the treatment of hAD-MSCs with SDF-1, western blotting was performed to measure the levels of the key proteins in the PI3K/Akt and ERK1/2 signalling pathways. A significant increase in the phosphorylation of Akt was induced by SDF-1 (*P* < 0.01; Fig. 8a, c). However, the level of phosphorylated ERK1/2 was not increased significantly after SDF-1 treatment (*P* > 0.05; Fig. 8a, b). The level of phospho-Akt increased significantly after SDF-1 treatment, while AMD3100 or LY294002 pretreatment significantly reduced the increased level of phospho-Akt induced by SDF-1 (*P* < 0.01; Fig. 8d, e).

Based on these results, the binding of SDF-1 to CXCR4 phosphorylates Akt and activates the PI3K/Akt signalling pathway, and the SDF-1/CXCR4 and PI3K/Akt signalling pathways are synchronously activated in hAD-MSCs by SDF-1 in vitro. The PI3K/Akt signalling pathway might be a downstream chemotactic homing signal in hAD-MSCs that is potentially activated by the SDF-1/CXCR4 axis.

### The PI3K/Akt signalling pathway is involved in the migration of hAD-MSCs mediated by the SDF-1/CXCR4 axis

Cells were pretreated with the inhibitor LY294002 before SDF-1 treatment to explore the effects of the PI3K/Akt signalling pathway on the migration of hAD-MSCs mediated by the SDF-1/CXCR4 axis.

**Fig. 9:**
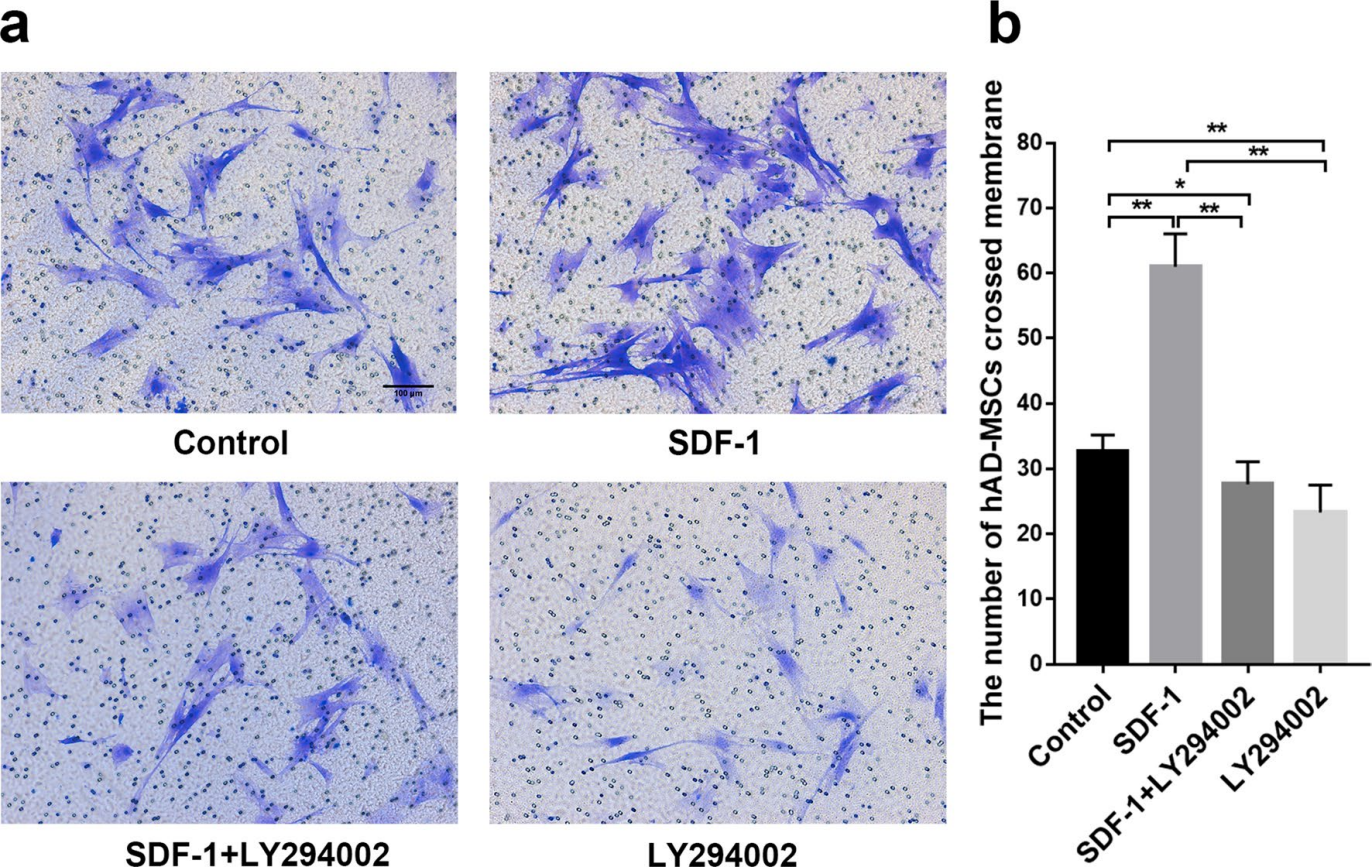
Effects of the PI3K/Akt signalling pathway on the migration of hAD-MSCs mediated by the SDF-1/CXCR4 axis. The migration of hAD-MSCs was tested using the Transwell migration assay (**a**), and the number of migrated cells was counted (**b**) in the control, SDF-1, SDF-1 + LY294002 and LY294002 groups under a microscope (×100). Representative images are shown. *N* = 6, *P < 0.05 and **P < 0.01. Scale bars = 100 μm

Compared to the control group, the number of hAD-MSCs passing through the membrane was significantly greater in the SDF-1 group (*P* < 0.01; Fig. 9a, b). Compared to the SDF-1 group, the number of hAD-MSCs passing through the membrane was significantly reduced by LY294002 pretreatment in the SDF-1 + LY294002 group (*P* < 0.01; Fig. 9a, b).

Therefore, the PI3K/Akt signalling pathway regulates the migration of hAD-MSCs mediated by the SDF-1/CXCR4 axis.

### Inhibition of the PI3K/Akt signalling pathway reduces hAD-MSC homing in vivo

We further confirmed whether the PI3K/Akt signalling pathway is involved in the homing of systemically transplanted hAD-MSCs to the ovaries of rats with chemotherapy-induced POI by pretreating cells with the inhibitor LY294002 before transplantation.

**Fig. 10:**
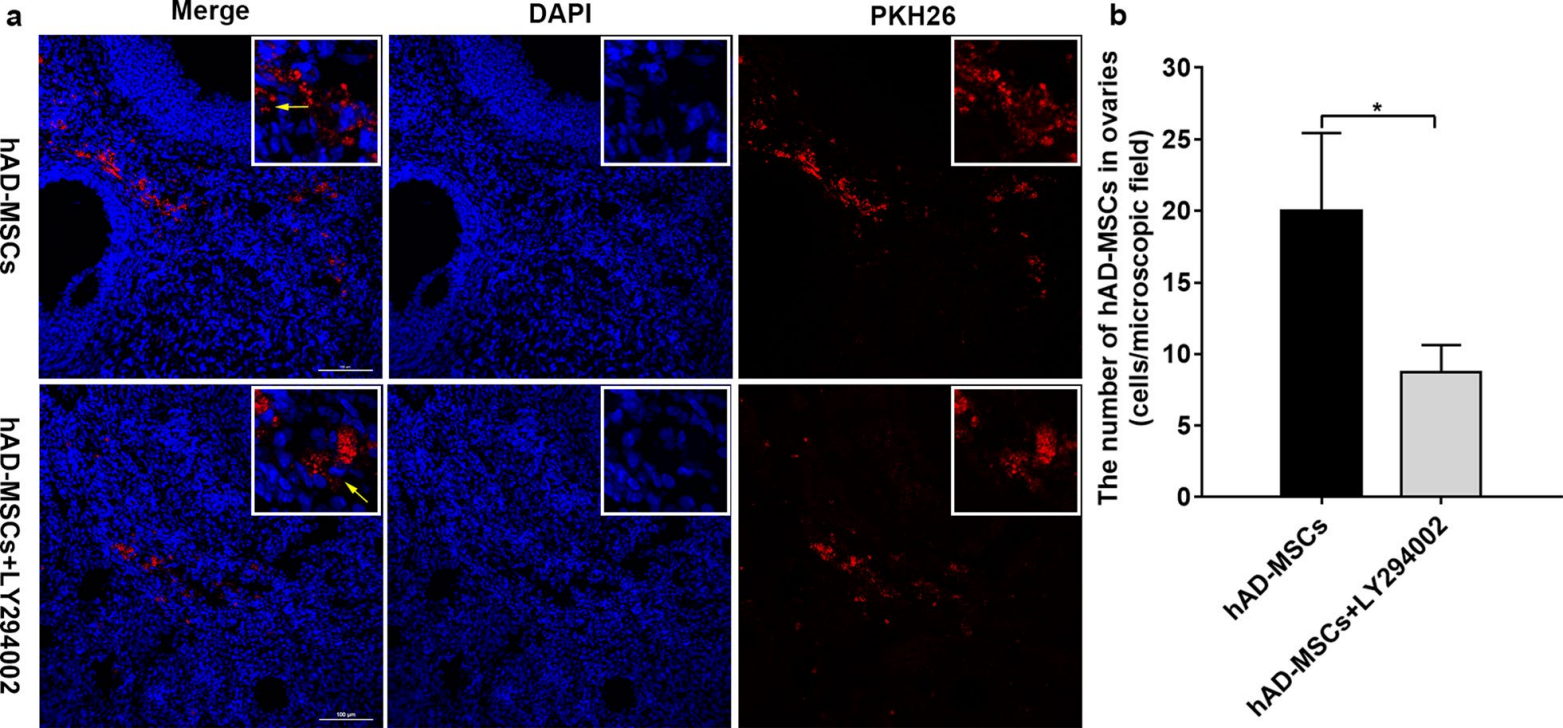
Inhibition of the PI3K/Akt signalling pathway reduces hAD-MSC homing in vivo. **a** Transplanted PKH26-labelled hAD-MSCs were observed in ovaries at 24 h after cell transplantation in the hAD-MSCs and hAD-MSCs + LY294002 groups using confocal microscopy (×200 and ×800). **b** The number of hAD-MSCs was counted and compared in the hAD-MSCs and hAD-MSCs + LY294002 groups (*n* = 10). Representative images are shown. The yellow arrows indicate transplanted hAD-MSCs. *P < 0.05. Scale bars = 100 μm

The homing and location of transplanted PKH26-labelled hAD-MSCs in the ovaries of POI rats were traced at 24 h after cell transplantation. PKH26-labelled cells with punctate red fluorescent signals were recruited and located in the interstitium of ovaries from rats with POI after transplantation in the hAD-MSCs and hAD-MSCs + LY294002 groups (Fig. 10a). No punctate red fluorescent signals were observed in the control and POI groups, consistent with our previously published articles [8, 11] (data not shown). A quantitative assessment of the number of hAD-MSCs in ovaries revealed that ovaries from the hAD-MSCs and hAD-MSCs + LY294002 groups contained 20.00 ± 17.22 and 8.70 ± 6.08 cells/microscopic field, respectively (*n* = 10; Fig. 10b). The intensity of punctate red fluorescent signals and the number of hAD-MSCs in the hAD-MSCs + LY294002 group were significantly lower than those of hAD-MSCs in the hAD-MSCs group (*P* < 0.05; Fig. 10a, b).

The inhibition of the PI3K/Akt signalling pathway reduces the homing of systemically transplanted hAD-MSCs to the ovaries of rats with chemotherapy-induced POI, and the PI3K/Akt signalling pathway might be involved in the homing of hAD-MSCs mediated by the SDF-1/CXCR4 axis in vivo.

## Discussion

As shown in the present study, the SDF-1/CXCR4 axis partially mediates the migration and homing of systemically transplanted hAD-MSCs to the ovaries of rats with chemotherapy-induced POI, and the PI3K/Akt signalling pathway might be involved in the migration and homing of hAD-MSCs mediated by the SDF-1/CXCR4 axis.

POI is caused by various factors, including chemotherapy, which results in multiple sequelae and has consequences on the health of women. POI is irreversible and currently incurable. Regenerative medicine studies have suggested that MSC transplantation may represent an effective treatment method for POI [3–5]. Our previous studies have also documented that some systemically intravenously transplanted hAD-MSCs home to the ovaries of rats with chemotherapy-induced POI, reduce ovarian injury and improve ovarian function [8, 38]. The migration and homing of MSCs to the injured tissue are essential for them to fulfil their functions in tissue regulation and repair. The ovary is the primary target organ injured by the toxic effects of cyclophosphamide used in clinical chemotherapy [39], which induces follicular loss, vascular damage, GC apoptosis, and tissue fibrosis in ovaries and causes POI [40, 41]. Thus, cyclophosphamide was chosen to establish the rat model of POI in our study. Interestingly, after hAD-MSC transplantation, no or few PKH26-labelled hAD-MSCs were observed in the liver, heart, brain, kidneys, and lungs, and most PKH26-labelled hAD-MSCs homed to the ovaries in rat chemotherapy-induced POI models [8], which indicates the tissue-specific homing of injected hAD-MSCs to the injured tissue in vivo. However, the cellular mechanisms that direct the migration and homing of hAD-MSCs to the ovaries of rats with chemotherapy-induced POI are incompletely understood.

Chemokines serve as strong chemoattractants for the homing of stem cells [24]. Some studies have found that chemokines released from tissue or endothelial cells and chemokine receptors expressed on MSCs may partially mediate active MSC homing to specific sites and subsequent retention in the tissue [16]. Chemokines that are upregulated in injured tissue and chemokine receptors expressed on MSCs might be involved in the efficiency of MSC homing [17, 27, 42].

SDF-1 is well known as a key regulator of the migration and homing of CXCR4-positive stem cells to the injured tissue [42, 43]. The SDF-1 concentration gradient is a driving force for stem cell migration [42]. SDF-1 is expressed at extremely low levels or is not originally detected in normal ovaries [44, 45]. However, after injury, the level of the chemokine SDF-1 increases in the tissue, which is critical for the chemotaxis of stem cells homing to the injured or ischaemic tissue [24, 27, 46–50]. SDF-1 was found to be the key cytokine regulating local inflammation and tissue repair by recruiting bone marrow stem cells to the sites of organ and tissue damage [51, 52]. Previous studies showed increased SDF-1 expression after myocardial infarction that mainly increased the retention of transplanted MSCs in the injured myocardium [53, 54]. Kitaori et al*.* detected an elevated SDF-1 level in the fracture site, which recruited MSCs homing to the site of injured bone and promoted bone repair [55]. It was reported that the expression level of SDF-1 was increased in the ovaries with chemotherapy-induced injury [56]. In our studies, very low SDF-1 levels were detected in the serum and ovarian tissue of normal rats in the control group, while the SDF-1 levels in the serum and ovarian tissue of rats with chemotherapy-induced POI were significantly increased. Thus, we speculated that the increased SDF-1 levels in the serum and ovaries of rats with POI might regulate the homing of transplanted hAD-MSCs from the systemic circulation to the ovaries, and further experiments were performed to test this hypothesis.

CXCR4, a specific chemotactic receptor for SDF-1, which is expressed in numerous types of adult stem cells [24], is a critical regulator of stem cell mobilization and recruitment. SDF-1 mobilizes stem cell migration into injured tissues by binding the CXCR4 receptor on stem cells [57, 58]. Although the molecular mechanisms that direct the migration and homing of MSCs are only partially understood [16], CXCR4 is proposed to play an important role in this process [35]. MSCs express functionally active CXCR4 at high levels [23, 24]. However, CXCR4 expression might vary in MSCs from different animal species and/or tissue types [59], and MSCs might lose the expression of homing molecules, such as CXCR4, during expansion in vitro [60, 61]. In our studies, hAD-MSCs were used at the third passage in the experiments, and the hAD-MSC population from different clones was proven to stably express the CXCR4 protein. The results confirm the abundant CXCR4 expression in hAD-MSCs, similar to CXCR4 expression in other types of MSCs [24, 35, 62].

As described above, SDF-1 is critical for the organ-specific chemotaxis and homing of stem cells to injured tissue [63], and these effects are mediated by SDF-1 binding to its receptor CXCR4 located on the surface of stem cells [64]. In our studies, SDF-1 levels in ovarian tissues and serum were significantly increased in rats with chemotherapy-induced POI, and hAD-MSCs expressed CXCR4. SDF-1 is a ligand and strong chemokine for CXCR4-expressing cells [35]. Thus, we further speculated that the SDF-1/CXCR4 axis might play a role in the migration and homing of hAD-MSCs to ovaries from subjects with chemotherapy-induced POI.

The homing of stem cells is associated with SDF-1 release from injured tissue as a chemoattractant and the SDF-1/CXCR4 axis plays an important role in the homing of stem cells [61, 65, 66]. SDF-1/CXCR4 interactions are implicated in a critical axis regulating stem cell trafficking and homing to the injured tissue [67]. The SDF-1/CXCR4 axis has been proven to direct the migration and homing of stem cells related to injury repair in many species and tissue types [24, 68]. The SDF-1/CXCR4 axis is also vital for modulating MSC migration and homing [35, 61]. Some studies found that the SDF-1/CXCR4 axis was a prerequisite for the homing of MSCs, and SDF-1 was a chemoattractive signal that regulated CXCR4^+^ MSC homing to injured tissues [42, 61, 69, 70]. In our studies, we detected strong chemoattractive activity of SDF-1 towards hAD-MSCs. The elevated SDF-1 level promoted the migration of hAD-MSCs in vitro, and the increased number of migrated hAD-MSCs induced by SDF-1 was significantly reduced by the CXCR4-specific antagonist AMD3100. Based on these results, SDF-1 induces the migration of hAD-MSCs in vitro, and the SDF-1/CXCR4 axis mediates the migration of hAD-MSCs induced by SDF-1. hAD-MSCs were pretreated with AMD3100 and systemically intravenously transplanted into POI rats to further explore whether the SDF-1/CXCR4 axis was involved in the migration and homing of hAD-MSCs to the ovaries of rats with chemotherapy-induced POI ovaries in vivo. Compared to the hAD-MSCs group, the number of hAD-MSCs homing to ovaries exhibiting POI was significantly decreased in the hAD-MSCs + AMD3100 group. Therefore, the SDF-1/CXCR4 axis is involved in the migration and homing of hAD-MSCs to the ovaries of rats with chemotherapy-induced POI. However, AMD3100 only partially reduced the number of hAD-MSCs homing to ovaries exhibiting POI, implying that the SDF-1/CXCR4 axis is not the only pathway involved in the migration and homing of hAD-MSCs in vivo. Thus, the SDF-1/CXCR4 axis partially mediates the migration and homing of systemically transplanted hAD-MSCs to the ovaries of rats with chemotherapy-induced POI.

The SDF-1/CXCR4 pathway is known as an upstream switch for many migration pathways. The binding of SDF-1 to CXCR4 activates multiple downstream signalling pathways in target cells, resulting in the phosphorylation of downstream effectors such as Akt, ERK1/2, focal adhesion kinase (FAK) and p38 [18], which in turn regulate various biological effects, including cell motility, chemotaxis and adhesion [18, 71, 72]. The PI3K/Akt signalling pathway, which is a downstream pathway of SDF-1/CXCR4, has been reported to regulate cell migration [26, 73]. Activation of PI3 kinase (PI3k) by SDF-1 in SDF-1/CXCR4-mediated chemotaxis is essential for cell migration, which subsequently results in the phosphorylation of downstream molecules such as Akt [34, 35]. PI3K/Akt is an important downstream pathway that regulates the migration of various stem and progenitor cells, including bone marrow MSCs, via the SDF-1/CXCR4 axis [19, 26, 34]. Studies have also shown that SDF-1/CXCR4-mediated chemotaxis may be driven by the activation of MAPK through ERK1/2 [19, 35]. According to Chen et al., SDF-1-mediated migration of cardiac stem cells is inhibited by blocking CXCR4, and the inhibitory effect involved a decrease in phospho-ERK1/2 levels [74]. Li et al. found that the SDF-1/CXCR4 axis mediates MSC migration, and the activation of the ERK 1/2 downstream signalling pathway induced by the binding of SDF-1 to CXCR4 is required for MSC migration [35]. Thus, PI3K/Akt and ERK1/2 signalling pathways were explored in this study to further investigate the molecular mechanisms involved in the migration and homing of hAD-MSCs to the ovaries of rats with chemotherapy-induced POI mediated by the SDF-1/CXCR4 axis.

The binding of SDF-1 to CXCR4 activated the PI3K/Akt signalling pathway, and the SDF-1/CXCR4 and PI3K/Akt signalling pathways were synchronously activated in hAD-MSCs by SDF-1. AMD3100 inhibited the SDF-1/CXCR4 axis and further indirectly inhibited the activation of the PI3K/Akt signalling pathway, which significantly reduced the SDF-1-induced migration of hAD-MSCs in vitro, while LY294002 directly inhibited the activation of the PI3K/Akt signalling pathway and significantly reduced the SDF-1-induced migration of hAD-MSCs in vitro. Cells were pretreated with the inhibitor LY294002 before transplantation to further confirm the effects of the PI3K/Akt signalling pathway on the homing of systemically transplanted hAD-MSCs to the ovaries of rats with chemotherapy-induced POI in vivo. Compared to the hAD-MSCs group, the LY294002 intervention significantly reduced the number of transplanted hAD-MSCs homing to ovaries exhibiting POI in the hAD-MSCs + LY294002 group. Therefore, we concluded that the PI3K/Akt signalling pathway plays an important role in facilitating the migration and homing of hAD-MSCs to the ovaries of rats with chemotherapy-induced POI along with the SDF-1/CXCR4 axis, and PI3K/Akt signalling might be the downstream pathway during hAD-MSC migration and homing to injured ovaries with POI. The PI3K/Akt signalling pathway might be involved in the migration and homing of hAD-MSCs mediated by the SDF-1/CXCR4 axis.

On the other hand, our studies also showed that the binding of SDF-1 to CXCR4 did not activate the ERK1/2 signalling pathway in hAD-MSCs, which is not consistent with some other studies [35, 74]. We consider that the activation of downstream signalling pathways by the SDF-1/CXCR4 axis related to cell migration might vary in stem cells derived from different animal species, tissue types and/or cell types. However, much more work is needed to further define these signalling pathways.

At present, local transplantation of MSCs is usually invasive, and the potential for minimally invasive delivery of MSCs via systemic infusion is still particular interesting. Systemic intravenous transplantation of MSCs for the treatment of POI has been shown to be efficacious and safe [3–5]. However, the low rate of cell homing, retention and survival after cell transplantation is a limitation of cell-based therapy for POI in current studies, and increasing the homing rate of systemically transplanted MSCs to ovaries in individuals with POI may increase the therapeutic efficacy [75, 76]. Moreover, enhancing MSC homing to a specific tissue is likely to significantly reduce the number of cells that is required to achieve a therapeutic effect on a disease and might provide better outcomes for patients [77]. As shown in our previous studies, systemic transplantation of hAD-MSCs partially reduces ovarian injury and improves ovarian function in rats with chemotherapy-induced POI [8]. However, the homing rate of systemically transplanted hAD-MSCs to ovaries exhibiting POI was low, which might limit the efficiency of MSCs for POI treatment [38]. In the present study, the CXCR4 antagonist blocked the SDF-1/CXCR4 axis and reduced the number of hAD-MSCs homing to ovaries in rats with POI, which consistently reduced their efficacy in POI treatment. Thus, a better understanding of the underlying molecular mechanisms that direct the migration and homing of hAD-MSCs to ovaries with chemotherapy-induced POI might be beneficial to studies exploring new method to increase the MSC homing rate and improve MSC transplantation efficacy, particularly in clinical applications. Our studies provide detailed evidence that the SDF-1/CXCR4 axis partially mediates the migration and homing of systemically transplanted hAD-MSCs to the ovaries of rats with chemotherapy-induced POI, and the PI3K/Akt signalling pathway might be involved in the migration and homing of hAD-MSCs mediated by the SDF-1/CXCR4 axis. These findings may provide new evidence to improve our understanding of the molecular mechanisms involved in the migration and homing of hAD-MSCs to the ovaries of individuals with POI, and the SDF-1/CXCR4 axis might be a potential target to explore for increasing the MSC homing rate and improving the efficacy of MSC transplantation for POI treatment, which will be explored in our subsequent studies.

## Conclusions

In conclusion, the SDF-1/CXCR4 axis partially mediates the migration and homing of systemically transplanted hAD-MSCs to the ovaries of rats with chemotherapy-induced POI. Systemic transplantation of hAD-MSCs partially reduces ovarian injury and improves ovarian function in rats with chemotherapy-induced POI, and blocking the SDF-1/CXCR4 axis with a CXCR4 antagonist reduces the number of hAD-MSCs homing to the ovaries of rats with POI and further reduces their efficacy in POI treatment. The PI3K/Akt signalling pathway, which is synchronously activated with the SDF-1/CXCR4 axis, plays a crucial role in the migration and homing of transplanted hAD-MSCs to the ovaries of rats with chemotherapy-induced POI, implying that the PI3K/Akt signalling pathway might be involved in the migration and homing of hAD-MSCs mediated by the SDF-1/CXCR4 axis. These findings may provide new evidence to improve our understanding of the molecular mechanisms involved in the migration and homing of hAD-MSCs to the ovaries of individuals with POI. This study describes a novel mechanism of hAD-MSC homing to the ovaries of rats with chemotherapy-induced POI, and the SDF-1/CXCR4 axis seems to be a potential target for improving MSC transplantation efficacy in POI treatment by attracting systemically transplanted MSCs to the injured ovaries of subjects with POI.

## Data Availability

All data and materials are available in the manuscript.

## Abbreviations

POI: Premature ovarian insufficiency
MSCs: Mesenchymal stem cells
hAD-MSCs: Human amnion-derived mesenchymal stem cells
BM-MSCs: Bone marrow-derived mesenchymal stem cells
SDF-1: Stromal cell-derived factor-1
CXCR4: CXC chemokine receptor 4
ERK1/2: P44/p42 extracellular signal-regulated kinases
PI3K: Phosphatidylinositol-3-kinase
L-DMEM: Dulbecco’s modified eagle medium low-glucose
FBS: Fetal bovine serum
CCK-8: Cell counting kit-8
AMH: Anti-Müllerian hormone
E2: Estradiol
FSH: Follicle-stimulating hormone
DAPI: 2-(4-Amidinophenyl)-6-indolecarbamidine dihydrochloride
OD: Optical density
SD: Sprague–Dawley
HE: Hematoxylin and eosin
PBS: Phosphate-buffered saline
ELISA: Enzyme-linked immunosorbent assay
ADM: Adipogenic differentiation medium
ODM: Osteogenic differentiation medium
CDM: Chondrogenic differentiation medium
VEGF: Vascular endothelial growth factor
VEGFR2: Vascular endothelial growth factor receptor 2
IS: Immunoreactivity score
ISCT: International society of cell therapy
GCs: Granulosa cells
FAK: Focal adhesion kinase
